# Introduction of *Cistanche phelypaea* fatty acids as a new natural neurotrophic supplement by evaluating its effects in normal and Alzheimer’s diseased rats

**DOI:** 10.1038/s41598-025-26267-8

**Published:** 2025-12-01

**Authors:** Doha H. Aboubaker, Ahmed A. A. Elsayed, Ahmed El-Gohary, Ibrahim A. El-Garf, Bassant M. M. Ibrahim

**Affiliations:** 1 Medicinal and Aromatic Plants Department, Pharmaceutical and Drug Industries Institute, National Research Centre, Dokki, Giza, Egypt; 2 Botany and Microbiology Department, Faculty of Science, Cairo University, Giza, Egypt; 3 Department of Pharmacology, Medical Research and Clinical Studies Institute, National Research Centre, Dokki, Po: 12622, Giza, Egypt

**Keywords:** Alzheimer model, *C. phelypaea*, Central nervous system booster, Polyunsaturated fatty acids, Biochemistry, Biological techniques

## Abstract

The incidence of the neurodegenerative disease “Alzheimer’s“(AD) is expected to reach up to 2.1 billion people aged 60 years old by 2050. Unsaturated fatty acids have been implicated in both protective and detrimental roles in AD. However, there is currently limited understanding of how varying levels of disease pathology in the brain influence the abundance of these fatty acid species. Within this framework, the present study aims to estimate the effect of fatty acids extracted from *Cistanche phelypaea* (*C. phelypaea*) on rats’ memory and behaviour. The fatty acids (FAs) extracted from the wild C. *phelypaea* were identified using GLC. Most of the FA were unsaturated. Oleic acid was the predominant fatty acid, followed by palmitic and linoleic acids. The study was conducted in two parallel arms, the first arm investigated the effect of *C. phelypaea* FAs on normal rats, and the second arm investigated its effect *on Alzheimer’s disease rats* and involved the following groups of rats: normal & untreated AD groups, reference group: donepezil (0.75 mg/kg), & *C. phelypaea* groups (100 and 200 mg/kg), all treatments were given concomitantly with AlCl_3_ (172.5 mg/kg) to rats, by oral gavage for 3 successive weeks, followed by alternating Y-maze & grid floor activity cage tests, then the levels of acetylcholine esterase (AchE), β-amyloid and tau protein were measured in rats’ sera of rats. Finally, histopathological examination of rats’ brains was done. In both arms, *C. phelypaea*, the number of movements in the grid floor activity cage was compared to normal and untreated AD groups. In the second arm, both doses reduced the level of AchE; the high dose only reduced β-amyloid compared to untreated AD. The results were consistent with the histopathologic results. *C. phelypaea* FAs had promising effects on brain health.

## Introduction

Over-exposure to aluminium (Al) in cosmetics, kitchen tools, and paints^1^ leads to its accumulation in the brain, causing inflammation and damage to the frontal cerebral cortex and the hippocampus, which is responsible for cognition and memory^2^. AL salts are rapidly absorbed via the oral route and can cross the blood-brain barrier easily, which enhances brain tissue inflammation, which is directly proportionate to frequency and amount of exposure^3^.

Neurodegenerative diseases like Alzheimer’s disease (AD) are due to damage to the frontal cerebral cortex and the hippocampus. Its incidence is approximately 5% between ages 65 and 74 years old and reaches up to 33.3% of the population aged 85 years or more^4^. It is expected to affect up to 2.1 billion people aged 60 years old by 2050^5^. Besides ageing, other factors contribute to the incidence of the disease, such as genes, stress, and environmental pollution, especially with AL, atherosclerosis and trauma. The hypotheses of AD development are the cholinergic hypothesis, formation of beta amyloids hypothesis, tau protein hyper-phosphorylation hypothesis, oxidative stress and inflammation hypotheses^6^. Affection of parts of the limbic system, which occurs in cases of AD, is manifested by impaired short-term memory, indifference, aggression, apathy and loss of motivation^7,8^.

Advancements have been achieved in the creation and licensure of disease-modifying medicines and supplements for symptomatic treatments of neuropsychiatric symptoms of Alzheimer’s disease during nearly two decades of vigorous pharmacologic and drug development efforts^9^.

“Desert hyacinths” refer to a fascinating group of parasitic plants in the genus Cistanche, which comprises over 20 recognized species commonly found in arid environments and coastal dunes, where they parasitize the roots of shrubs. Among these, several Cistanche species have been valued for centuries as sources of traditional herbal medicine. Therefore, the present study aims to estimate the effect of *C. phelypaea* FAs, which is proposed as a new natural product that is supposed to possess boosting capabilities on mental activities in normal rats. It also aims to evaluate the protective effect of *C. phelypaea* FAs against Alzheimer’s disease in rat models mimicking Alzheimer’s disease in humans.

## Materials and methods

### Materials

#### Plant material

The whole wild plants of *C. phelypaea* (L.) Cout (513 CAI) were collected from Wadi Araba, Eastern Desert, during March 2021, Latitude (N 29.6845986), Longitude (E 31.4184590). The plants were kindly authenticated by Prof. Dr. Ibrahim A. El-Garf, Faculty of Science, Cairo University, Egypt. Voucher specimens have been deposited in the Cairo University Herbarium.

#### Animals

Thirty male Wister albino rats weighing 150–175 g were used in the present study. The rats were obtained from the animal house colony of the National Research Centre in Dokki, Giza, Egypt. The rats were kept in metal cages at room temperature 22 ± 3 °C, with 55 ± 5% humidity, fed standard rat chow and allowed free water access. Experimental procedures were performed in accordance with “The Guide for Care and Use of Laboratory Animals and the Animal Procedures” and followed the recommendations of “All experimental protocols were approved by the National Institutes of Health Guide for Care and Use of Laboratory Animals (18–156).

#### Drugs, chemicals, and kits for biochemical assays

Donepezil hydrochloride (Aricept; Pfizer Inc, New York, NY, USA), Formaldehyde (Al-Gomhoria Company for medicines and medical supplies, Egypt), Diethyl ether (Sigma Aldrich, USA), High-quality kits for measuring the levels of aspartate aminotransferase (AST), alanine aminotransferase (ALT), urea, creatinine in rats’ sera were bought from Biodiagnostic Company, Egypt. Elisa kits reagents for assaying acetylcholine esterase (AchE), beta-amyloid and tau protein, in rat sera were of the highest purity grade commercially available.

#### Behaviour stress tests apparatus

Grid floor activity cage, locally made wooden Y-maze.

#### Tested substance

*C. phelypaea* FAs given in 100 and 200 g /kg.

### Methods

#### Total lipids extraction

Plants were dried and powdered. The total lipids were extracted from 400 g dried *C. phelypaea* by petroleum ether using Soxhlet equipment (1 × 500 ml, 12 h). This Soxhlet extraction method followed Ramluckan et al. (2014)^10^ protocol. The solvent was evaporated using a rotary evaporator under reduced pressure.

#### GLC analysis of fatty acid Methyl esters

The process described by Metcalfe et al. (1966) involved heating fatty acids in a 10% methanol solution of borontrifluoride to transesterify them into methyl esters. A Shimadzu GC-14 A with a flame ionization detector and a C-R4AX chromatopac integrator (Kyoto, Japan) was used to identify fatty acid methyl esters (FAME). The split value with a ratio of 1:40 was employed, and the carrier gas (helium) flow rate was 0.6 mL/min. A 30 m × 0.25 mm × 0.2 g film thickness Supelco SP M-2380 (Bellefonte, PA, USA) capillary column was used to inject a 1 µL sample. The temperature of the injector and detector was fixed at 250 °C. The starting column temperature was set at 100 °C, and it was programmed to rise by 5 °C per minute to 175 °C and remain there for 10 min. Next, it increased by 8 °C per minute to 220 °C and remained there for 10 min. To make identification easier, the retention durations of the samples were compared to those of an authentic standard mixture (Sigma, St. Louis, MO, USA; 99% purity specific for GLC) and run on the same column under the same conditions. Without using a correction factor, the peak areas of each fatty acid’s methyl ester and methyl nonadecanoate were compared in order to quantify each one.

#### Study design: the study duration was three weeks and Ran in two parallel arms as follows

*First study: effect of C. phelypaea* FAs *on normal rats* 1st group: Five male albino Wister rats served as a normal group, which were allowed free access to water and ad libitum without any intervention. 2nd group: Five male albino Wister rats were given 200 mg/kg of *C. phelypaea* by oral gavage using gastric tubes.

*Second study: Effect of C. phelypaea* FAs *on Alzheimer’s disease rats* 1st group: The same normal rats of the effect of *C. phelypaea* on normal rats were employed in this study. 2nd group: Untreated Alzheimer’s disease group, which served as a positive control group. Alzheimer’s disease was induced by oral gavage of 172.5 mg/kg of aluminium chloride (AlCl_3_) daily for three successive weeks^11^. 3rd group: Donepezil (0.75 mg/kg) was given concomitantly with AlCl_3_ (172.5 mg/kg) to rats by oral gavage^12^ and served as the reference group. 4th and 5th groups: Treated groups were given *C. phelypaea* concomitantly with AlCl_3_ (172.5 mg/kg) to rats by oral gavage in doses of 100 and 200 mg/kg^13^.

After three successive weeks of administration of *C. phelypaea* to normal rats, as well as giving treatment concomitant with administration of AlCl_3_ for brain insult, assessments of the effects of *C. phelypaea* were done in comparison to normal, positive control and reference groups by the following investigations:

#### Behaviour stress tests

Assessment of cognition and short-term memory for both studies was done by alternating Y-maze test according to the method of Gharib et al.^14^. In both studies, the assessment of the psychological state of rats by recording their activity in the grid floor activity cage was mentioned by Abd El-Halim et al.^15^ and followed the method described by Pavic et al.^16^.

#### Biochemical assays

Assays for evaluating the effects of subchronic administration of a previously proven safe dose of *C. phelypaea* (200 mg/kg)^13^, on normal rats’ livers and kidneys included measuring the levels of AST, ALT, urea, and creatinine in sera according to the methods of^17–20^.

Assaying the levels of AchE, β-amyloid and tau protein in sera of rats of all groups in both studies was done. The levels of AchE, β-amyloid and tau protein were measured using ELISA kits and followed the manufacturer’s guidelines.

#### Histopathological examination

Autopsy samples were taken from the brain, liver & kidney of rats in the first study and only from the brains of rats in the second study, then fixed in 10% formol saline for twenty-four hours. Washing was done in tap water, and then serial dilutions of alcohol (methyl, ethyl and absolute ethyl) were used for dehydration. Specimens were cleared in xylene and embedded in paraffin at 56 degrees in a hot air oven for 24 h. Paraffin beeswax tissue blocks were prepared for sectioning at 4 microns thickness by rotary LEITZ microtome. The obtained tissue sections were collected on glass slides, deparaffinized, and stained by hematoxylin & eosin stain^20^ for examination under the light electric microscope.

### Statistical analysis

Statistical analysis for comparing means + standard errors (SE) of all groups in both studies was carried out by using Graph Pad Prism software (version 9, USA). A two-tailed T test was used for processing data of the study of the effect of *C. phelypaea* on normal rats; the significance value was *p* < 0.05. While the one-way analysis of variance “ANOVA” test followed by the “Tukey–Kramer Post hoc test” was used to perform the statistical analysis of the study of the effects of *C. phelypaea and* donepezil on AD rats, the significance value was *p* < 0.0001.

## Results and discussion

In order to be able to manage cognitive disturbance associated with AD, there is a distinct need to discover new disease-modifying agents, as choline esterase inhibitors that are the most commonly used therapeutics, have some bothering side effects as nausea, vomiting and diarrhea^21^, also Alves et al.^22^ reported that the use of either lecanemab or donanemab which are monoclonal antibodies used as an add-on anti-amyloid for management of AD symptoms, caused brain swelling which can hasten brain atrophy^22^.

Our study involved the use of *C. phelypaea* FAs as a new neurotrophic natural supplement for compacting signs and symptoms of AD in rat models mimicking sufferers of AD, and for enhancing behavioral performance in normal rats. This was achieved after studying the phytochemical profile of *C. phelypaea*, and was based on studying the fatty acid profile of the total lipids found in *C. phelypaea* as presented in Table 1, which provides a summary of information regarding the qualitative and quantitative makeup of fatty acids.

**Table 1:** Relative percentages of fatty acids in *C. phelypaea FAs*.

Fatty acids	Percent	Fatty acids	Percent
Capric acid	3.82	Oleic acid	28.72
Myristic acid	3.94	Linoleic acid	18.60
Palmitic acid	25.15	Linolenic acid	1.53
Palmetoleic acid	5.24	Arachidic acid	0.82
Stearic acid	3.53	Total unknown compounds	8.65

The FA profile of the total lipids found in *C. phelypaea* shows that the lipids are an excellent source of linoleic acid, which is nutritionally necessary. The predominant fatty acid isoleic acid, which is followed by palmitic acid and linoleic acid. An expanding body of literature highlights the role of polyunsaturated fatty acids as potential regulators of inflammatory responses in the central nervous system, with a specific focus on microglial activation Layé et al.^23^. Therefore, *C. phelypaea* extract, based on its components of FAs was introduced in the present study as a new supplement for modifying brain functions in rats susceptible to neurodegenerative disorders.

**Fig. 1:**
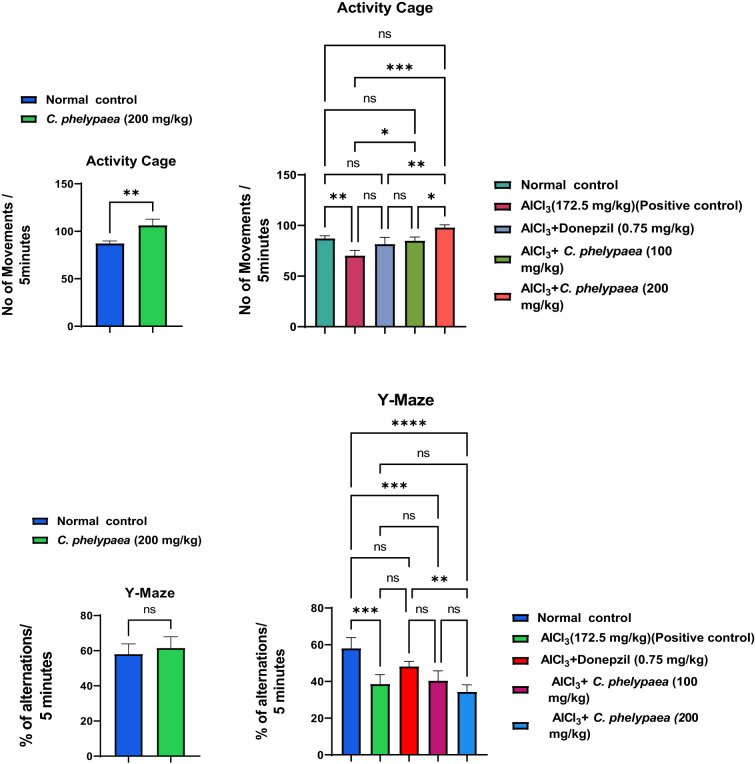
Results are expressed as means of movements across the grid floor activity cage and % of alternations in the Y-maze /5 min ± SE. *N* = 5. Comparisons done using two tailed t test in the first study, for comparison between normal and *C. phelypaea FAs group* (200 mg/kg) only. Significance at P value < 0.05. Comparisons done using ANOVA, followed by Tukey Kramer’s test for multiple comparisons, in the second study, for comparison between normal, untreated AlCl_3_ (172.5 mg/kg) (positive control group), AlCl_3_ + donepezil (0.75 mg/kg), AlCl_3_ + *C. phelypaea* FAs (100 mg/kg) and AlCl_3_ + *C. phelypaea* FAs (200 mg/kg) groups. Significance at P value < 0.0001. SE = standard error, N = number of rats/group, ns = non-significant, *= the number of asterisks denotes the degree of significance between groups.

**Fig. 2:**
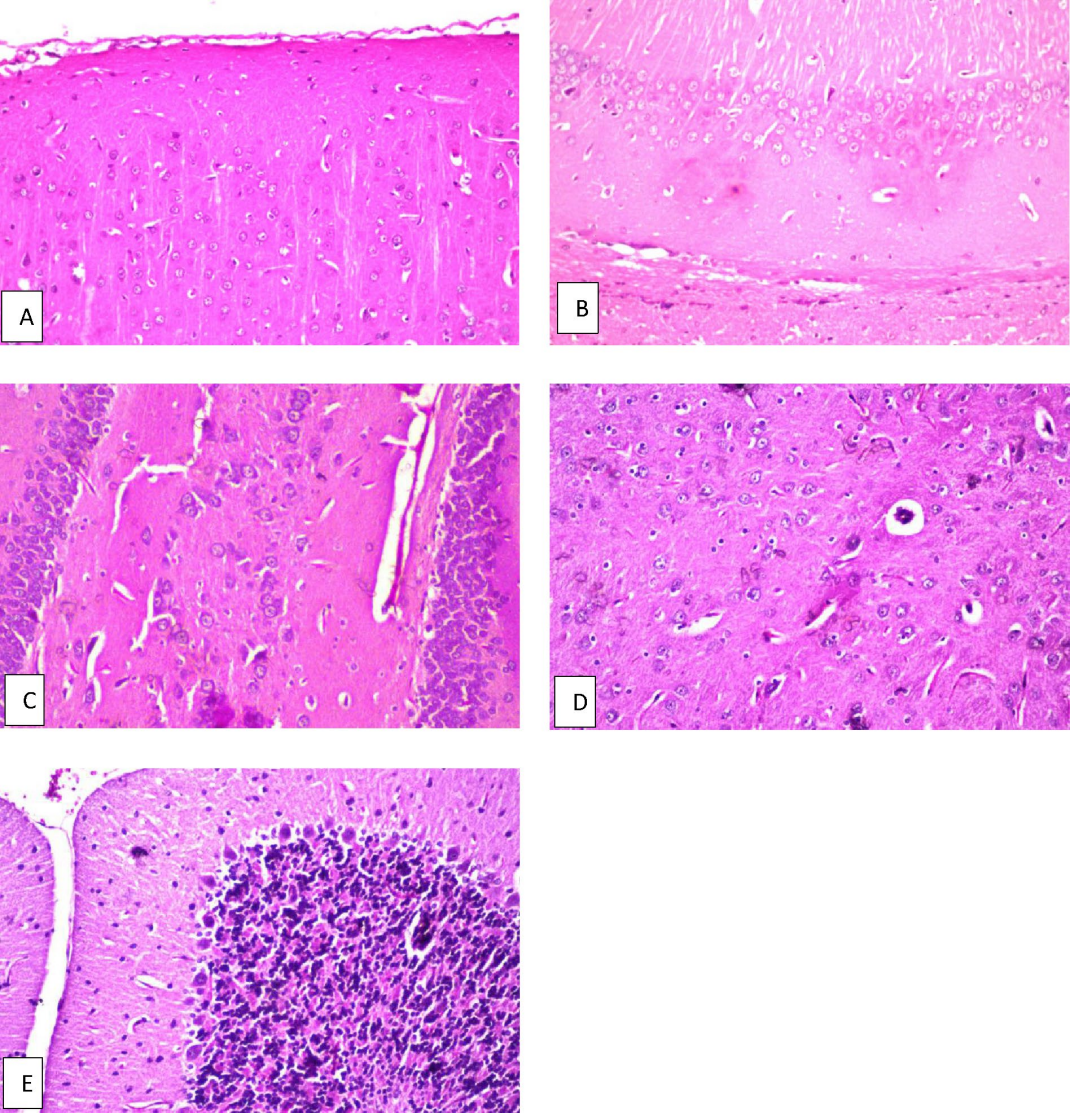
Brain of normal rat. **a** Cerebral cortex, **b** hippocampus subiculum, **c** fascia dentata, **d** striatum and **e** cerebellum, all of them show no histopathological alteration and the normal histological structure of the neurons was recorded.

The rats were classified into: A normal group, that was allowed free access to water and diet without any intervention. This group was also used as the normal group in the second study to and the results of the experimental procedures of the first and second studies were compared to it. The results of the behavioral activity of this group, concerned with assessing the psychological state of rats by using the grid floor activity cage and assessing the cognitive abilities of rats by using the Y-maze, were presented in Fig. 1. The results of the effect of *C. phelypaea* FAs on the liver and kidney function tests in the normal group that received *C. phelypaea* FAs only were exhibited in Table 2, also the biochemical assay of markers associated with the neurological activity of the same group were mentioned in Table 2 for the first study The histopathologic description of brain specimens of this group was normal, as described in Fig. 2.

**Table 2:** Effect of *C. phelypaea FAs* (200 mg/kg) on normal rats.

Parameter	Group	
	Normal group	*C. phelypaea* (200 mg/kg)
AST (U/L)	33.3 ± 3.55	65.0 ± 3.60^@^
ALT (U/L)	21.7 ± 0.97	26.5 ± 0.56^@^
Urea (gm/dl)	26.5 ± 1.29	27.5 ± 0.70
Creatinine (gm/dl)	0.8 ± 0.02	0.8 ± 0.03
AchE (ng/ml)	2.2 ± 0.03	2.3 ± 0.11
β amyloid (pg/ml)	242.2 ± 5.14	266.0 ± 19.41
Tau protein (ng/ml)	1.1 ± 0.00	1.2 ± 0.03

Results are expressed as means of levels of AST, ALT, Urea, Creatinine, AchE, β amyloid and tau protein in serum ± SE. *N* = 5. Comparisons were done using two tailed t test. Significance at P value < 0.05. ^@^Significantly different from Normal group.

The effect of *C. phelypaea* FAs, when given orally in a dose of 200 mg/kg for three successive weeks to normal rats, that weren’t exposed to any hazardous conditions affecting brain functions, *in the first study of this work*, shows significant increase in the number of movements across the grid floor activity cage when compared to the normal group that didn’t receive the *C. phelypaea* which owes to its high contents of FAs (Fig. 1). This indicates that *C. phelypaea* FAs improved the psychological state of rats and motivated them to perform better in the grid floor activity cage, compared to rats that were left without any intervention and were just allowed free access to diet and water. Yet, there wasn’t any significant difference in the Y-maze test between the normal group that received *C. phelypaea* and the normal group that didn’t receive it (Fig. 1).

The results of the behavior stress test performed using grid floor activity cage and Y-maze were confirmed by biochemical assaying of the levels of Ach-E, βamyloid and tau protein as there weren’t any significant differences between their levels in both groups (Table 2). Also, histopathologic examination of the brains of rats from both groups showed normal structure of neurons in both groups (Figs. 2, 5).

**Fig. 3:**
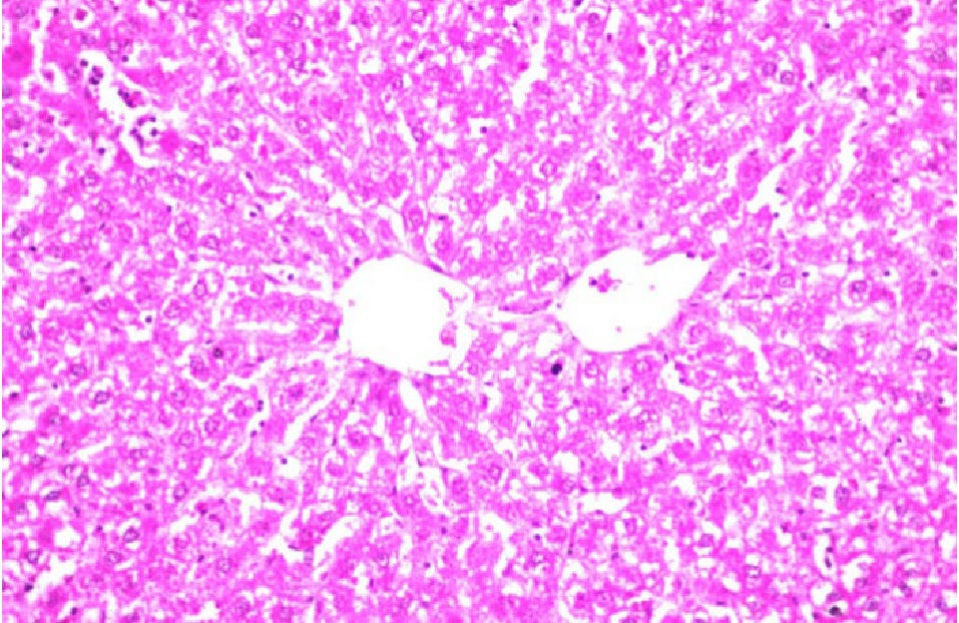
Liver of normal rat given *C. phelypaea* FAs (200 mg/kg) alone for three weeks shows degenerative change in the hepatocytes all over the parenchyma.

**Fig. 4:**
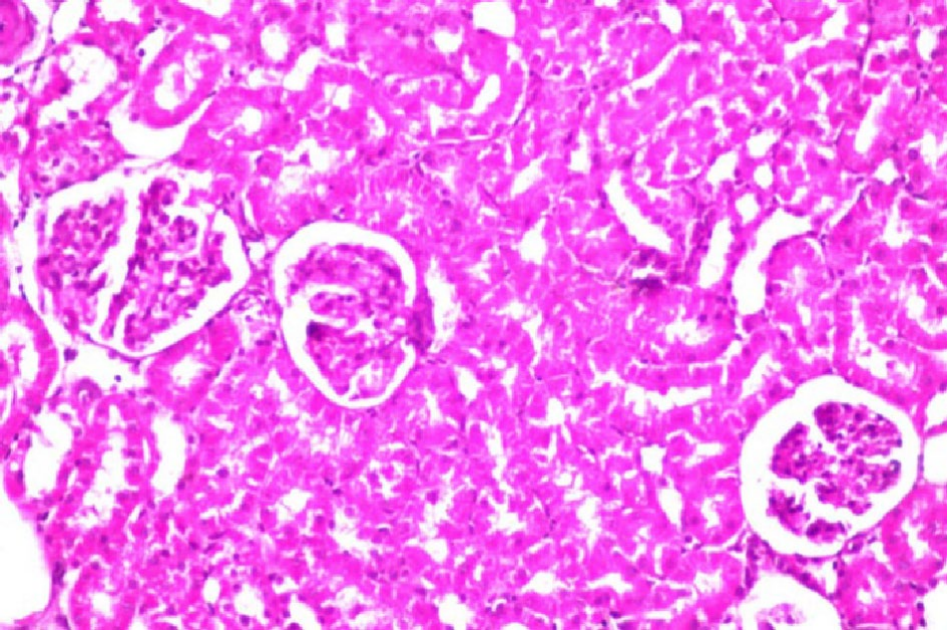
Kidney of normal rat given *C. phelypaea* FAs (200 mg/kg) alone for three weeks shows no histopathological alteration.

Additionally, the effect of *C. phelypaea* FAs (200 mg/kg) given orally for three successive weeks, on the liver and kidneys of normal rats in the first study, was also evaluated. Unfortunately, the liver functions AST & ALT were significantly elevated in the group receiving *C. phelypaea* FAs (200 mg/kg), compared to the normal group (Table 2). Moreover, the histopathologic examination of the livers of the *C. phelypaea* group showed degenerative change in the hepatocytes (Fig. 3). These findings indicate that *C. phelypaea* FAs (200 mg/kg), should be used cautiously in normal individuals with continuous monitoring of hepatic enzymes, and should be avoided in cases diagnosed with underlying hepatic disease. On the other hand, the urea and creatinine levels of the *C. phelypaea* group in the first study, were nearly the same as the normal group that received only diet and water (Table 2). Also, histopathologic examination of kidneys of the *C. phelypaea*, group revealed no abnormalities (Fig. 4).

Regarding *the second study of this work*, which focused on the effects of *C. phelypaea* FAs (100 & 200 mg/kg), when it was given concomitantly with AlCl_3_ (172.5 mg/kg) orally for three successive weeks, to study its potential protective effects against AD, the observed results were as follows:

A) Effect of oral administration of AlCl_3_ for three successive weeks without concomitant intake of any treatment whether *C. phelypaea* FAs or donepezil: It caused a significant reduction in the number of movements of rats across the grid floor activity cage, denoting that it caused marked deterioration in the psychological state of rats compared to the normal group and all treated groups (Fig. 1). However, in the Y-maze test, it caused high significant reduction in the % of alternations of rats between the arms of the Y-maze, when compared only to the normal rats that weren’t exposed to AlCl_3_ (172.5 mg/kg) administration (Fig. 1), this implies that AlCl_3_ markedly affected cognition in rats, which mimics symptoms of AD in humans. AlCl_3_ (172.5 mg/kg), also significantly increased the levels of Ach-E when compared to the normal and all treated groups, that were given *C. phelypaea* FAs (100 & 200 mg/kg) and donepezil (0.75 mg/kg), concomitantly with AlCl_3_ (Fig. 6). Moreover, it increased significantly the level of β amyloids when compared to the normal, donepezil and *C. phelypaea* FAs (200 mg/kg) groups but showed no significant difference when compared to the group that received *C. phelypaea* FAs (100 mg/kg). Additionally, it increased the level of tau protein significantly when compared to the normal group, the donepezil and the *C. phelypaea* FAs (100 mg/kg) group, without a significant difference when compared to the *C. phelypaea* FAs (200 mg/kg) group (Table 3). Admission of AlCl_3_ (172.5 mg/kg) orally to rats in the second study without concomitant treatment, caused histopathologic changes in their brains in the form of nuclear pyknosis and degeneration in all neurons in the cerebral cortex, additionally, the fascia dentata showed nuclear pyknosis and degeneration in few neurons, and the striatum showed nuclear pyknosis and degeneration in most of the neurons, on the other hand, the hippocampus subiculum and the cerebellum showed no histopathological alteration (Fig. 6).

**Fig. 5:**
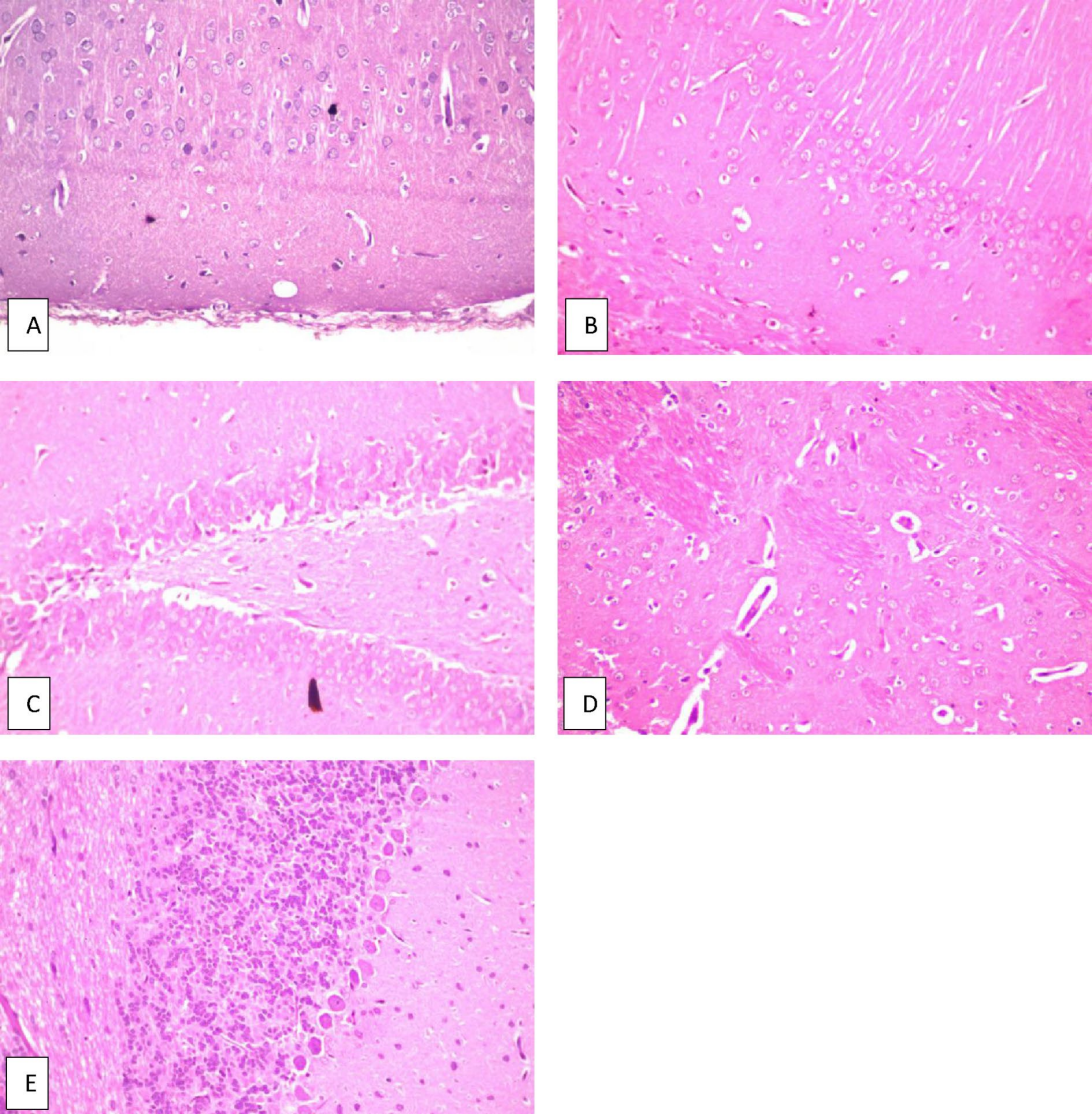
Brain of normal rat given *C. phelypaea* FAs (200 mg/kg) alone. **a** Cerebral cortex, **b** hippocampus subiculum, **c** fascia dentata, **d** striatum and **e** cerebellum, all of them show no histopathological alteration.

**Table 3:** Protective effects of *C. phelypaea FAs* (100 & 200 mg/kg) compared to donepezil (0.75 mg/kg) against alzheimer’s disease induced in rats by using AlCl_3_ (172 mg/kg).

Group	Parameter		
	AchE (ng/ml)	β amyloid (pg/ml)	Tau protein (ng/ml)
Normal group	2.24 ± 0.04	242.20 ± 5.14	1.11 ± 0.00
Positive control (AlCl_3_ 172.5 mg/kg)	2.82 ± 0.06^@^	380.40 ± 8.57^@^	1.32 ± 0.01^@^
Donepezil (0.75 mg/kg)	2.22 ± 0.06*	221.90 ± 2.91*	1.15 ± 0.01*
*C. Phelypaea FAs* (100 mg/kg)	2.50 ± 0.04*	404.50 ± 7.71^@&^	1.16 ± 0.02*
*C. Phelypaea FAs* (200 mg/kg)	2.47 ± 0.05*	345.40 ± 4.11^@^*^&$^	1.26 ± 0.02^@&$^

Results are expressed as means of levels of AchE, β amyloid and tau protein in serum ± SE. *N* = 5. Comparisons were done using ANOVA, followed by Tukey Kramer’s test. Significance at P value < 0.0001. ^@^Significantly different from normal group, *significantly different from positive control group, ^&^significantly different from Donepezil group, ^$^significantly different from *C. phelypaea* (100 mg/kg) group.

**Fig. 6:**
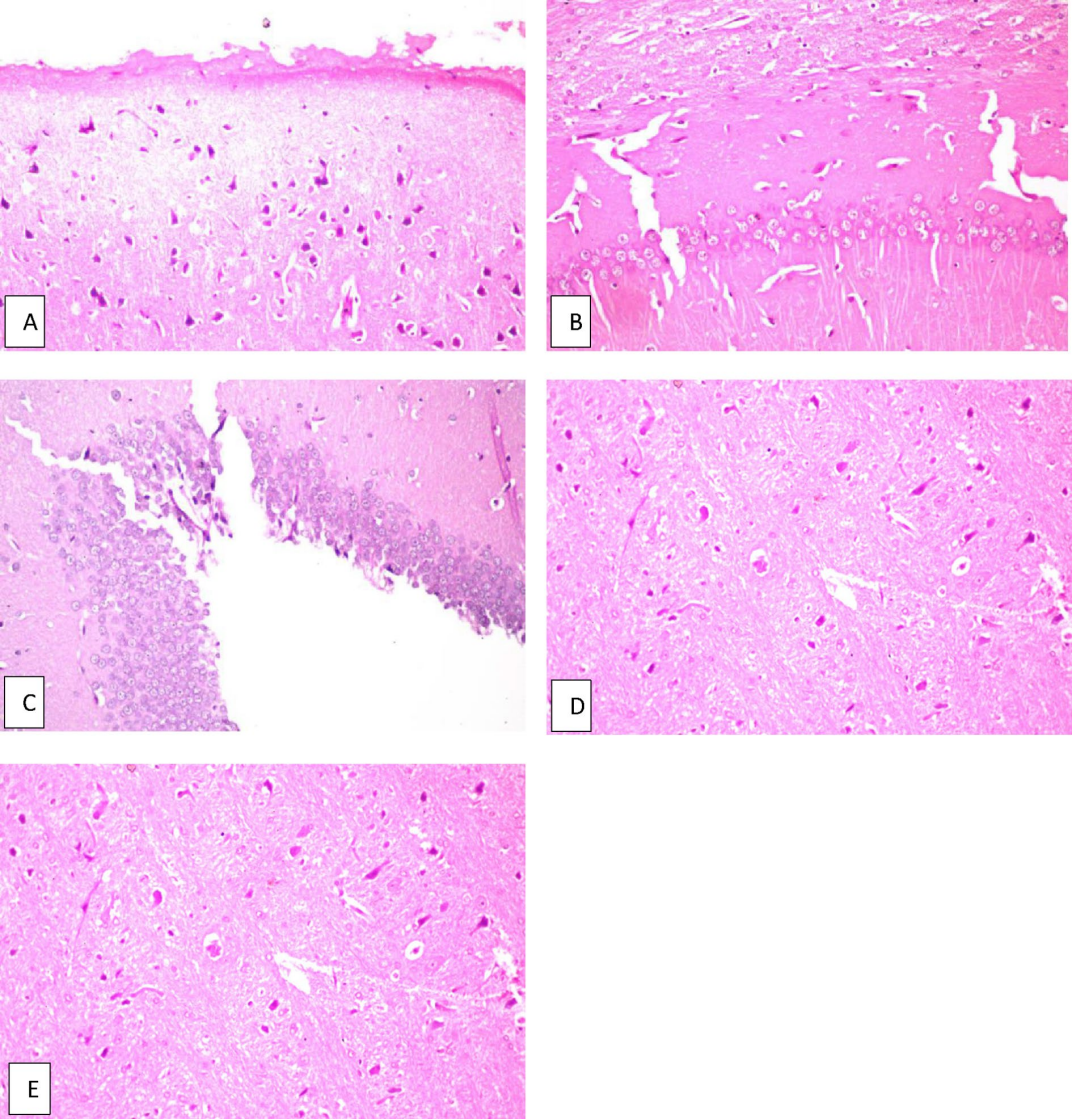
Brain of untreated AD rat (positive control given AlCl_3_ (172.5 mg/kg) without treatment). **a** Cerebral cortex shows nuclear pyknosis and degeneration were observed in all neurons, **b** hippocampus subiculum shows no histopathological alteration, **c** fascia dentata shows nuclear pyknosis and degeneration in few of the neurons, **d** striatum shows nuclear pyknosis and degeneration in most of the neurons, **e** cerebellum shows no histopathological alteration.

These signs and symptoms of AlCl_3_ insult to rat brain that mimic AD in humans were the same as those reported by Aboubaker et al.^11^ in their study. The histopathologic changes in the fascia dentata are common in old age and in AD, as stated by Crain and Burger^24^, which is the same picture in our study. Maya et al.^25^ revealed in their study that intoxication with aluminium reduces the antioxidant capacity and increases free radical generation and inflammatory activity, in addition to changes in brain protein configuration^25^. Moreover, Alasfar and Isaifan^26^, stated that excessive accumulation of aluminium reduces ATP production and causes apoptosis, due to changes in mitochondria^26^. The previous neurologic changes lead to Alzheimer’s incidence due to neurodegeneration^3^, and amyloid deposition which causes neuronal death^27^. Disturbed cognition in rats of the positive control (untreated AlCl_3_) in the current study can be attributed to apoptotic neuronal changes in the cerebral cortex as a result of deposition of AlCl_3_^28^.

The results of the biochemical assay in the current study reveal high levels of β amyloid and tau proteins due to intake of AlCl_3_, and are associated with symptoms of AD, which are similar to ageing in normal rats, these results can be explained by protein denaturation in neurons caused by Al, according to what was mentioned in the study of Massand et al.^3^.

B) Potential protective effects of treatment with *C. phelypaea* FAs (100 & 200 mg/kg) compared to normal and positive control groups as well as to donepezil (0.75 mg/kg) group, which acts as reference group: In the present work treatments were given orally for three successive weeks, concomitantly with AlCl_3_ (172.5 mg/kg), to investigate their possible protective effects against development of AD or their ability to slow down the progression of AD induced by AlCl_3_.

The results of the effects of *C. phelypaea* FAs (100 & 200 mg/kg) on the psychological state and cognition of rats were presented in Fig. 1, treatment with both doses didn’t show any significant change in the number of movements of rats across the grid floor activity cage when compared to the normal group. On the other hand, both doses exhibited a significant increase in the number of movements of rats across the grid floor activity cage when compared to the positive control group. Additionally, the high-dose FAs group showed a significant increase in the number of movements of rats across the grid floor activity cage when compared to the reference drug group and the low-dose FAs group, but the low-dose FAs group showed a nearly equivalent effect to the reference drug group (donepezil).

While, the results of the effects of treatment, when investigated by using the Y-maze weren’t very promising, as none of the three treatments showed significant changes in the % of alternations of rats along the Y-maze arms, when compared to the positive control group, moreover, the effects of both doses of the FAs showed a significant reduction in the % of alternations of rats when compared to the normal group, additionally, the high dose FAs group showed a significant reduction in the % of alternations of rats when compared to the reference drug group, but there weren’t any significant changes between both high and low doses of the FAs groups.

The previous results suggest that treatment with the *C. phelypaea* FAs (100 & 200 mg/kg), improves the psychological state of rats subjected to induction of neurological insult by the use of AlCl_3_. However, it doesn’t have noticeable effects on cognition of the same group of rats, its effect in the low dose was the same as the reference drug effect.

Regarding the biochemical assay exhibited in Table 3, C. *phelypaea* FAs (100 & 200 mg/kg) and donepezil significantly decreased the level of Ach-E, without significant differences either between the three groups and each other or between the three groups and the normal group. Also, both doses of *C. phelypaea* FAs, as well as donepezil, significantly reduced the levels of β amyloid compared to the positive control group. However, the levels of β amyloid in both groups were significantly higher than the normal and donepezil groups, and the level was significantly higher in the low dose group than the high dose group. Treatment with the low dose of *C. phelypaea* FAs, as well as with donepezil, significantly reduced the tau protein levels compared to the positive control group. Additionally, there weren’t any significant differences between the low dose of the FAs group and the donepezil group also no significant difference was detected between the donepezil group and the low dose of the FAs group on one side and the normal group on the other side. However, the high dose of the *C. phelypaea* FAs exhibited a significant increase in the level of tau protein compared to the normal, donepezil and low dose of *C. phelypaea* FAs groups, yet there wasn’t any significant difference between the level of tau protein in the positive control group and the *C. phelypaea* FAs high dose group.

**Fig. 7:**
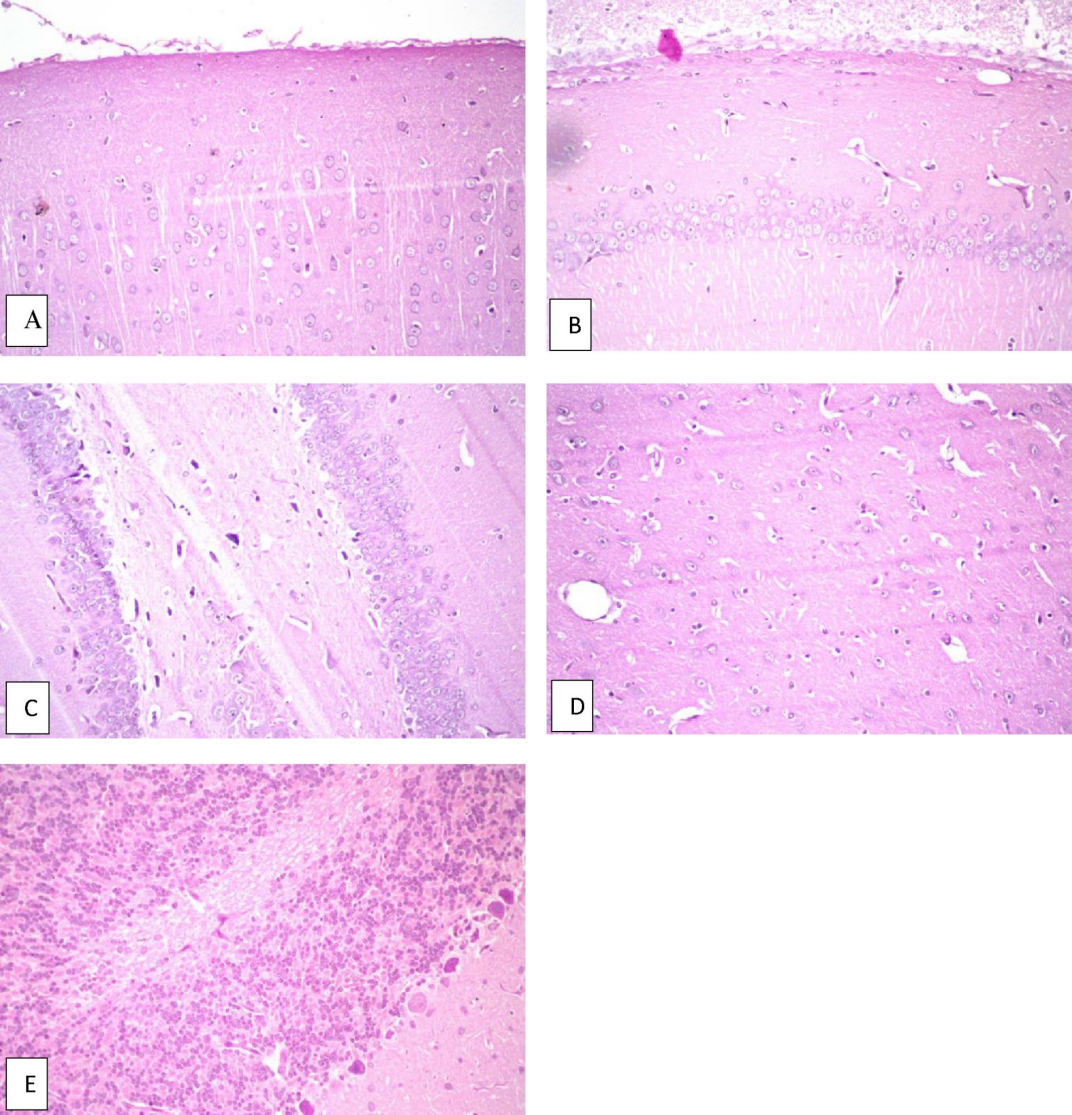
Brain of AD rat treated with donepezil (0.75 mg/kg) (reference group). **a** Cerebral cortex and **b** hippocampus subiculum, show no histopathological alteration, **c** fascia dentata shows nuclear pyknosis and degeneration in few of the neurons, **d** striatum shows diffuse gliosis in between the intact neurons, **e** cerebellum shows no histopathological alteration.

**Fig. 8:**
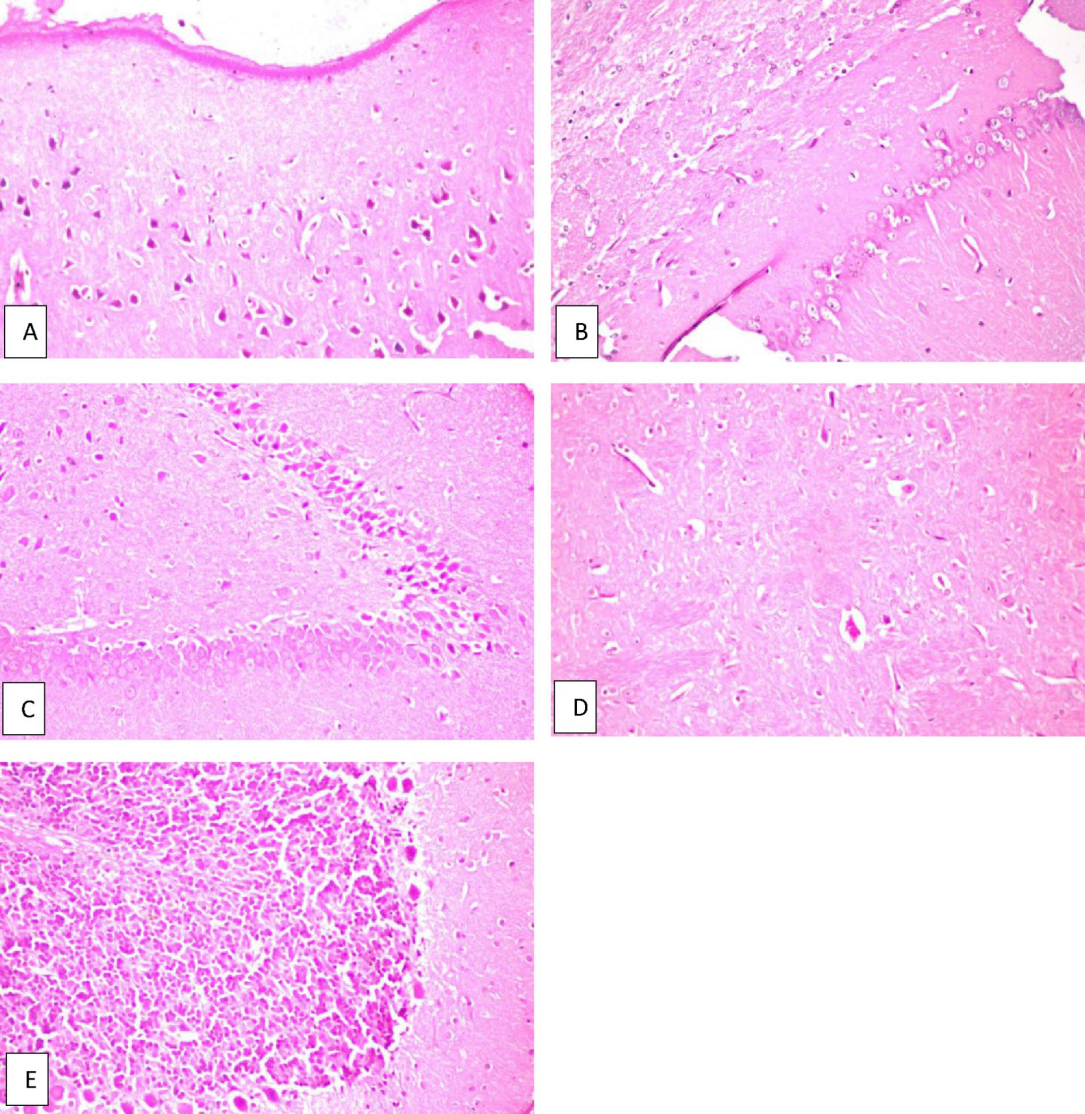
Brain of AD rat treated with *C. phelypaea* FAs (100 mg/kg). **a** Cerebral cortex shows nuclear pyknosis and degeneration in most of the neurons, **b** hippocampus subiculum shows no histopathological alteration, **c** fascia dentata shows nuclear pyknosis and degeneration in some few neurons, **d** striatum shows nuclear pyknosis and degeneration in some neurons, **e** cerebellum shows no histopathological alteration.

**Fig. 9:**
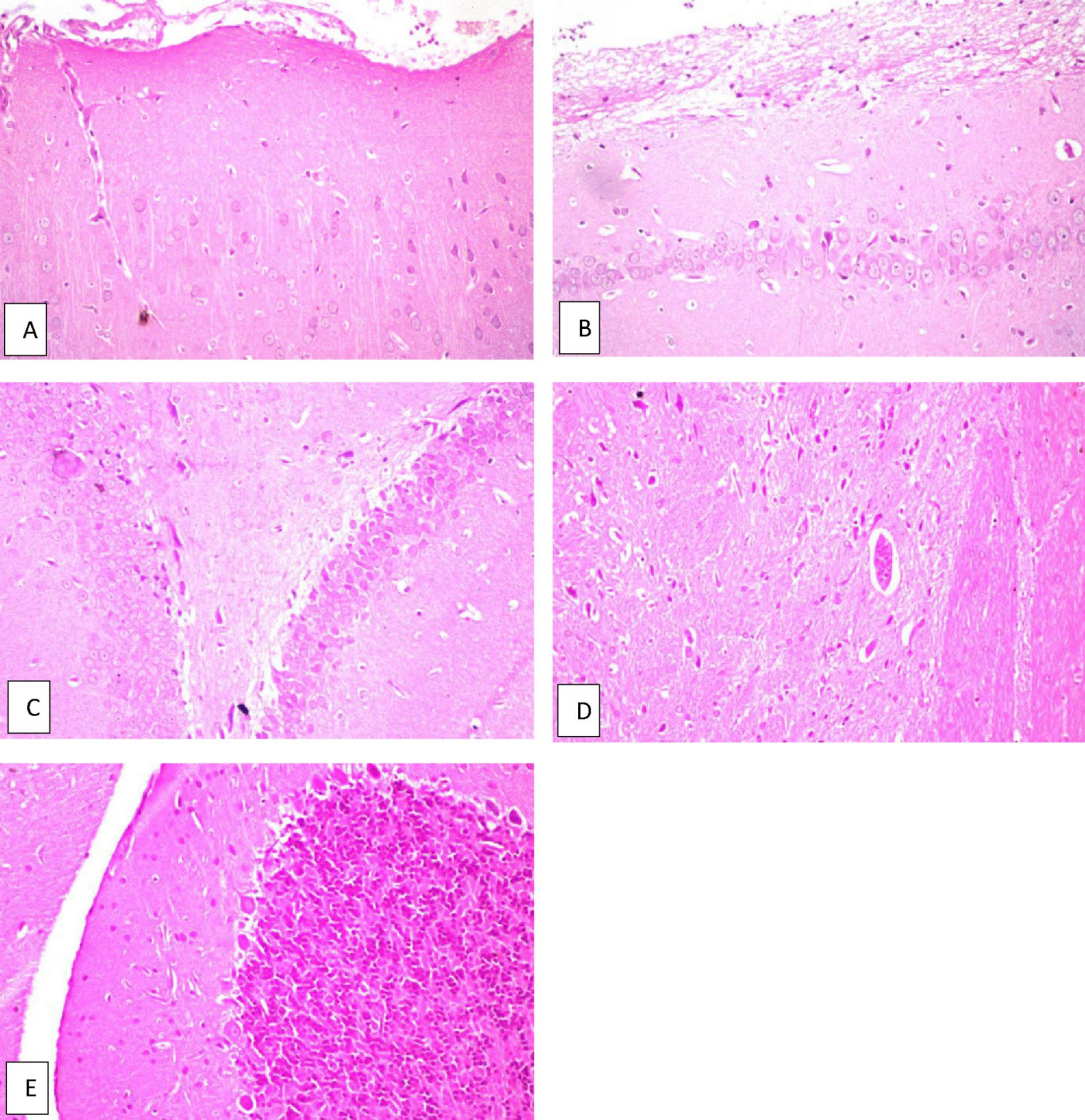
Brain AD rat treated with *C. phelypaea* FAs (200 mg/kg). **a** Cerebral cortex, **b** hippocampus subiculum and **c** fascia dentata show no histopathological alteration, **d** striatum shows nuclear pyknosis and degeneration in some of the neurons with congestion of the blood vessels, **e** cerebellum shows no histopathological alteration.

The biochemical assay results were confirmed by the histopathologic examination results of the brain specimens of rats, which were as follows: The group of rats that received donepezil (0.75 mg/kg) concomitantly with AlCl_3_ showed nuclear pyknosis and degeneration in few neurons of the fascia dentata, and diffuse gliosis in between the intact neurons of the striatum, with no histopathologic abnormalities in other parts of the brain (Fig. 7). Examination of the brains of rats treated with *C. phelypaea* FAs (100 mg/kg), showed nuclear pyknosis and degeneration in most of the neurons of the cerebral cortex, and showed nuclear pyknosis as well as degeneration in some few neurons of the fascia dentata and nuclear pyknosis and degeneration in some neurons of the striatum, while the hippocampus subiculum and the cerebellum showed no histopathological alteration (Fig. 8). The brains of rats treated with *C. phelypaea* FAs (200 mg/kg), showed nuclear pyknosis and degeneration in some neurons of the striatum with congestion of the blood vessels, but no other histopathologic abnormalities in other parts of the brain were detected (Fig. 9).

The outcome of treatment of rats susceptible to AD, with *C. phelypaea* FAs in the current study, is most probably due to its mechanism of action, that was explained by Zhou et al.^28^, who reported that *Cistanche* acted by reducing high levels of reactive oxygen species inside the mitochondria, increased the antioxidant capacity, and reduced the oxidative stress, thus inhibited neuro-inflammation via inhibiting the activity of the microglia involved in the inflammatory process. It also reduced tau proteins hyper-phosphorylation and accumulation of β amyloids, and it also promoted the reduction of AchE activity^29^, which is consistent with the results of our study. Thus, *C. phelypaea* FAs helped to keep acetylcholine (Ach) and noradrenaline at normal levels. *C. phelypaea* FAs may have activated the *N*-methyl-d-aspartate-receptor, which helped to keep synapses healthy and exhibited neuronal anti-apoptotic activity^29^, which is the mechanism thought to be responsible for memory formation^30^.

**Fig. 10:**
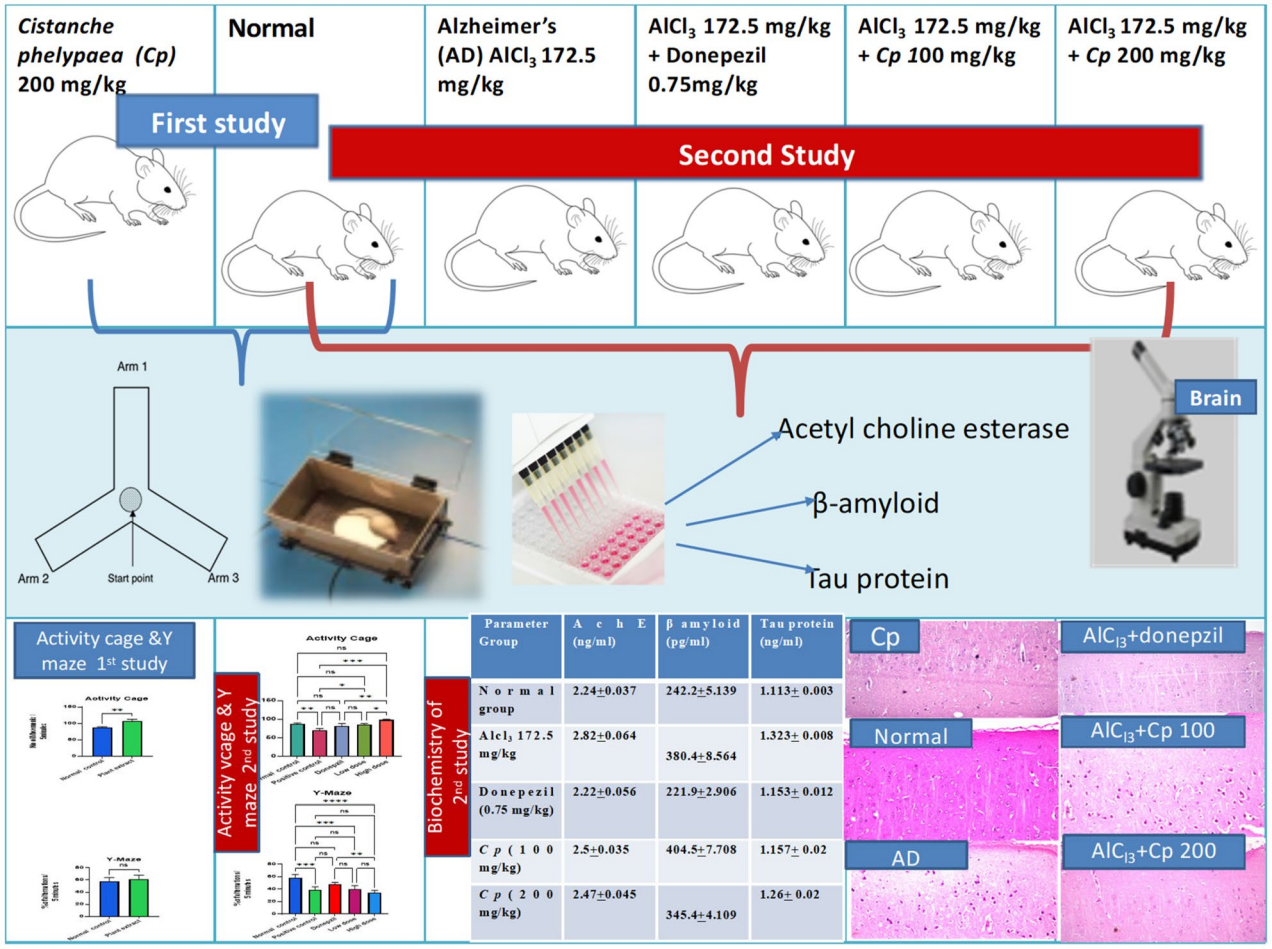
Schematic overview illustrates the study design and highlights the key results.

This mechanism of action was confirmed by the histopathology results in our study, it also explains the results of behaviour stress tests as illustrated in Fig. 10.

Previous studies have investigated the levels of unsaturated fatty acids (UFAs) in the brains of patients with Alzheimer’s Disease (AD). Nasaruddin et al.^29^ found that the abundance of 20 fatty acids increased in the Brodmann area 7 of patients with late-stage AD. Cunnane et al.^30^ analyzed fatty acids in both plasma and brain tissue samples, reporting decreased levels of esterified DHA in the AD group, particularly in phosphatidylserine in the middle frontal and superior temporal cortices.

Polyunsaturated fatty acids (PUFAs) can be broadly categorized into two groups: omega-3 and omega-6 fatty acids. Alpha-linolenic acid (omega-3) and linoleic acid (omega-6) are considered “parental” essential fatty acids, as they convert into long-chain UFAs, with arachidonic acid derived from linoleic acid and DHA and eicosapentaenoic acid (EPA) arising from linolenic acid. Amtul et al.^31^ conducted an in vitro study to assess the effects of linoleic acid and arachidonic acid—as well as omega-9 fatty acid, oleic acid, on the pathology of AD. They demonstrated that linoleic, arachidonic, and oleic acids could stimulate the polymerization of both tau and amyloid beta (Aβ)^31^. While this information and additional literature^32^ may suggest that oleic acid has pathogenic effects, a substantial body of literature also supports the notion that oleic acid offers protective effects against AD^33^. Specifically, Amtul et al. demonstrated that, oleic acid supplementation lowered secreted Aβ levels; they validated these findings in a transgenic mouse model on an oleic acid-rich diet^34^. The potential cognitive benefits of oleic acid supplementation in humans are underscored by the health properties of olive oil, which is abundant in oleic acid and is thought to protect against age-related cognitive decline and the onset of AD^35–39^. In a clinical trial, Martinez-Lapiscina et al.^38^ reported that patients randomized to an olive oil-rich diet exhibited better cognitive function compared to those on a control diet^38^.

**Fig. 11:**
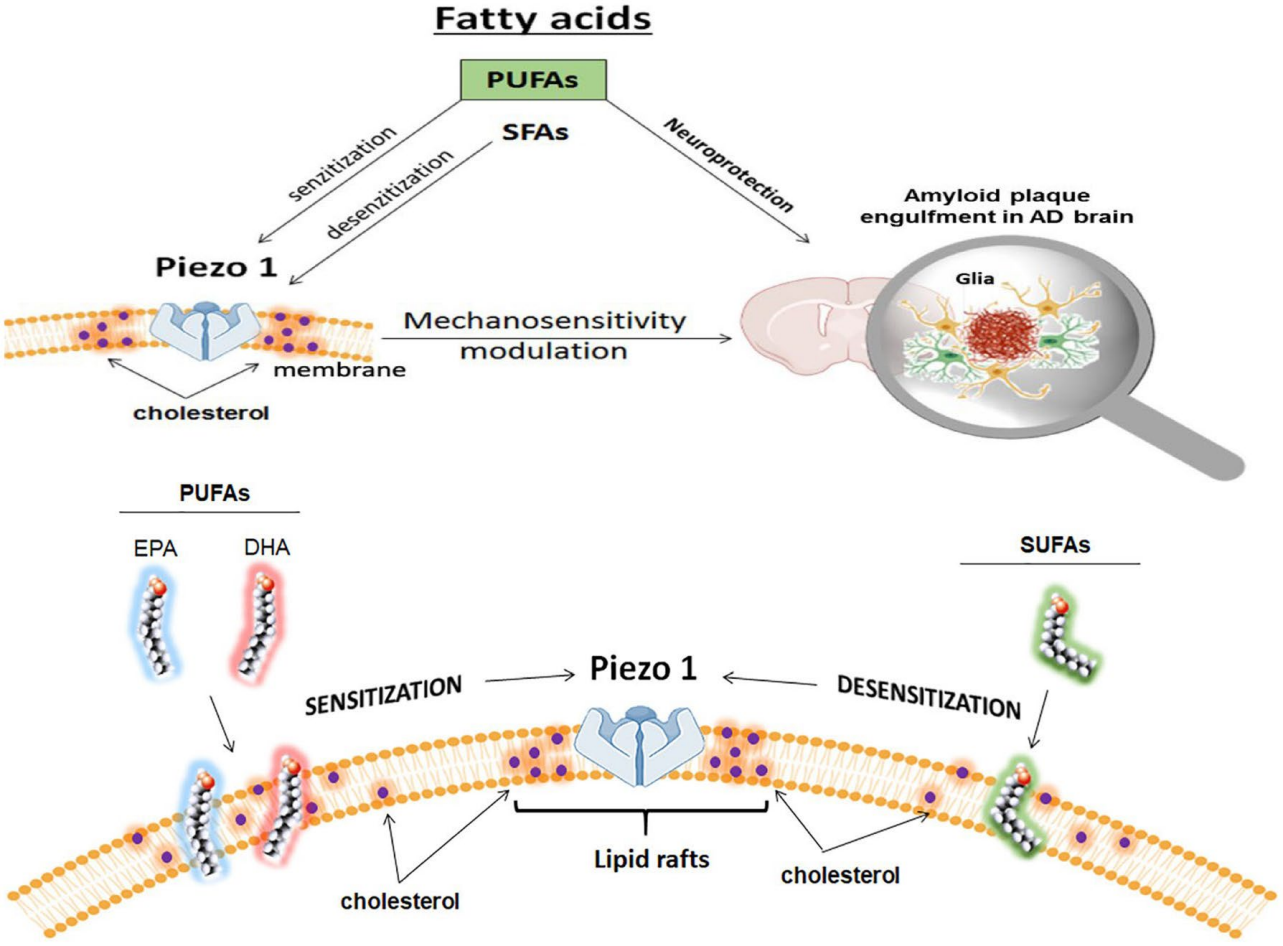
The schematic illustrates how fatty acids (FAs) regulate Piezo1 by modulating membrane elasticity. Piezo1 is situated within lipid raft areas enriched with cholesterol, while FAs influence the stiffness of non-raft regions of the membrane. Long-chain n-3 polyunsaturated fatty acids (PUFAs), such as DHA and EPA, enhance membrane elasticity, whereas saturated fatty acids (SFAs) increase membrane stiffness. Consequently, alterations in the non-raft regions of the membrane can affect the sensitivity of Piezo1. Moreover, PUFAs can influence microglial polarization, affect the encasement of amyloid plaques, and modify immune responses.

Interestingly, linolenic acid, the precursor to the predominant brain component docosahexaenoic acid (DHA) and eicosapentaenoic acid (EPA), has emerged as a potential novel protector of brain health. Its effects may be particularly relevant for carriers of the ApoE4 allele, as linolenic acid appears to function by enhancing the integrity of the blood-brain barrier (BBB). Additionally, dietary supplementation with DHA, has been shown to enhance cognitive performance in various animal studies of AD^39–42^. Previous human studies have also reported promising cognitive effects in individuals receiving DHA, EPA, or a combination of the two^43–45^ (mechanism illustrated in Fig. 11). The potential mechanisms by which DHA supplementation may influence AD include the modulation of beta-amyloid deposition in the brain^46^. The potentially protective effects of EPA in the brain may involve competitive inhibition of lipoxygenases (LOX), cyclooxygenases (COX), and cytochrome P450 (CYP450) against its omega-6 counterpart, arachidonic acid (ARA)^47−50^. The breakdown of ARA by COX and 5-LOX results in the production of pro-inflammatory prostaglandin E2 and leukotriene B4^47,48^. In contrast, the breakdown of EPA by these enzymes produces less potent pro-inflammatory products, such as prostaglandin E3 and leukotriene B5^47−50^.

Cistanche herbs and their primary chemical constituents exhibit promising neuroprotective effects against neurodegenerative diseases such as AD, Parkinson’s disease (PD), and cerebral ischemia-reperfusion injury. Cistanche herbs demonstrates significant therapeutic potential in the context of AD. It has been shown to enhance cognitive abilities in individuals with moderate AD, delay hippocampal atrophy, and reduce the expression levels of tau protein, tumor necrosis factor-alpha (TNF-α), and interleukin (IL)-1β. Furthermore, extracts of *C. tubulosa* have been found to mitigate β-amyloid (Aβ) 1-42-induced cognitive dysfunction in rats by preventing amyloid deposition and restoring cholinergic neuronal function^51^. It is noteworthy that *Cistanche* herbs also promot neuronal cell differentiation, neurite elongation, and synapse formation in the hippocampus, ultimately enhancing learning and memory functions in mice^52-54^. Therefore, FAs from *C. phelypaea* are promising anti-AD therapeutic agents. Further studies will help to clarify more in-depth mechanism.

## Conclusion

It can be deduced from the results of the current preclinical study that *C. phelypaea* FAs have a supporting and boosting effect on the central nervous system when given to normal rats as it boosts memory and improves the psychological state of rats. However, more studies regarding the posology of the FAs should be considered, especially in hepatic disorders.

Also, *C. phelypaea* FAs exhibited promising effects in protection against brain damage induced by AlCl_3_, but with variable degrees between low and high doses, that varied according to the parameter evaluated. The high dose improved rat motivation manifested by increased ambulation across the grid floor activity cage even better than the reference drug donepezil; this may be because the high dose reduced β amyloid, thus reducing neuro-inflammation and subsequently maintaining the brain structure as manifested by histopathologic examination, it also reduced AchE which increased Ach, thus helped to increase activity of rats. *C. phelypaea* FAs is a promising brain anti-ageing natural product, that can be used for protection against the development of AD, but with caution with hepatic morbidities.

## Data Availability

The datasets used in this investigation are all included in this publication, and upon justifiable request, they are also available from the corresponding author.
