# Author Correction: Introduction of *Cistanche phelypaea* fatty acids as a new natural neurotrophic supplement by evaluating its effects in normal and Alzheimer’s diseased rats

**DOI:** 10.1038/s41598-025-34156-3

**Published:** 2025-12-31

**Authors:** Doha H. Aboubaker, Ahmed A. A. Elsayed, Ahmed El-Gohary, Ibrahim A. El-Garf, Bassant M. M. Ibrahim

**Affiliations:** 1 Medicinal and Aromatic Plants Department, Pharmaceutical and Drug Industries Institute, National Research Centre, Dokki, Giza, Egypt; 2 Botany and Microbiology Department, Faculty of Science, Cairo University, Giza, Egypt; 3 Department of Pharmacology, Medical Research and Clinical Studies Institute, National Research Centre, Dokki, Po: 12622, Giza, Egypt

Correction to: *Scientific Reports* 10.1038/s41598-025-26267-8, published online 01 December 2025

The original version of this Article contained an omission in the name of Bassant M. M. Ibrahim, which was incorrectly given as Bassant M. M. Ibrahims.

The original Article has been corrected.

